# Perioperative immunomodulation with interleukin-2 in patients with renal cell carcinoma: results of a controlled phase II trial

**DOI:** 10.1038/sj.bjc.6603391

**Published:** 2006-10-10

**Authors:** T Klatte, A Ittenson, F-W Röhl, M Ecke, E P Allhoff, M Böhm

**Affiliations:** 1 Department of Urology, Otto-von-Guericke-University, Leipziger Straße 44, 39120 Magdeburg, Germany; 2 Institute of Immunology, Otto-von-Guericke-University, Leipziger Straße 44, 39120 Magdeburg, Germany; 3 Institute of Biometrics and Medical Informatics, Otto-von-Guericke-University, Leipziger Straße 44, 39120 Magdeburg, Germany; 4 Department of Urology, Städtisches Klinikum, Birkenallee 34, 39130 Magdeburg, Germany

**Keywords:** renal cancer, interleukin-2, immunotherapy, surgery, survival

## Abstract

We conducted a non-randomised controlled phase II trial to investigate the role of preoperative administration of interleukin-2 (IL-2) in patients with renal cell carcinoma undergoing tumour nephrectomy. A total of 120 consecutive patients were allocated alternately to the two study groups: perioperative immunomodulation with IL-2 (IL-2 group; *n*=60) and perioperative immunomonitoring without immunomodulation (control group; *n*=60). Patients from the IL-2 group received four doses of 10 × 10^6^ IU m^−2^ twice daily subcutaneously a week before operation followed by a daily maintenance dose of 3 × 10^6^ IU m^−2^ subcutaneously until a day before the operation. Parameters of cellular and humoral immunity (leucocytes, T-cell markers CD3, CD4, and CD8, B-cell marker CD19, monocyte marker CD14, natural killer (NK) cell markers CD16, CD56, and CD57, activation markers CD6, CD25, CD28, and CD69, progenitor cell marker CD34, as well as IL-2, IL-6, IL-10, soluble IL-2 receptor, IL-1 receptor antagonist, transforming growth factor-*β*1, and vascular endothelial growth factor) were measured in peripheral venous blood at various intervals. Interleukin-2-related toxicity was WHO grade 1 (24%), 2 (67%), and 3 (9%). In the postoperative period, T-cell markers, activation markers, and NK cell markers decreased, and IL-6 and IL-10 increased. However, all these alterations were significantly less accentuated in patients who had been pretreated with IL-2. Median follow-up was 40 months. Tumour-specific survival in the IL-2 group and the control group was 98 *vs* 81% after 1 year and 86 *vs* 73% after 5 years (*P*=0.04). A similar effect was found for progression-free survival. We conclude that IL-2 can be safely administered in the perioperative period and modulates immunological parameters. However, to validate the survival data, a larger randomised phase III trial is needed.

Renal cell carcinoma (RCC) accounts for 3% of all adult malignancies. Over 38 000 new cases and 12 000 deaths were estimated for 2006 in the United States (Jemal *et al*, 2006). Surgery is a mainstay of therapy for both localised and metastasised RCC. However, surgery is associated with considerable perioperative dysfunction of the immune system (Böhm *et al*, 2001). Also, circulating tumour cells are frequently found in patients with RCC (McKiernan *et al*, 1999; Ashida *et al*, 2000; Bilkenroth *et al*, 2001; Shimazui *et al*, 2004) and are more prevalent in the renal vein and after surgery (Ashida *et al*, 2000), possibly owing to surgery-associated dissemination of tumour cells into the blood circulation, and the presence of circulating tumour cells appears to be associated with a poor prognosis (Shimazui *et al*, 2004).

Considered an immunosensitive disease, RCC is being treated with various immunotherapeutic regimens both before and after surgery. So far, no survival benefit has been demonstrated (Pizzocaro *et al*, 2001; Clark *et al*, 2003; Messing *et al*, 2003; Atzpodien *et al*, 2005; Bex *et al*, 2006). In these studies, no immunotherapy was administered during the perioperative period when immunodysfunction is most pronounced and circulating tumour cells are most frequently detected.

On this background, we conducted a prospective controlled phase II trial to assess the effect of immunomodulation with interleukin-2 (IL-2) in the potentially critical perioperative period in patients with RCC undergoing tumour nephrectomy.

## PATIENTS AND METHODS

### Study objectives and patient eligibility

Patients with a renal tumour who were scheduled for radical nephrectomy at our institution were eligible for entry into this phase II trial. Each patient met the following eligibility criteria: (a) Eastern Cooperative Oncology Group performance status (ECOG PS) of ⩽2; (b) life expectancy >3 months; (c) age at least 18 years; (d) adequate bone marrow, hepatic, and renal function; and (e) negative pregnancy test for women of childbearing potential. Exclusion criteria were as follows: no histologically proven RCC; prior or synchronous malignancies; autoimmune diseases; pregnancy or lactation; uncontrolled infections; chronic debilitating diseases; positive HIV status; and the presence of contraindications to IL-2.

Informed consent was obtained from 120 consecutive patients. They were allocated alternately to the two study groups: perioperative immunomodulation with IL-2 (IL-2 group; *n*=60) and perioperative immunomonitoring without immunomodulation as a control group (control group; *n*=60). The study was approved by the local ethics committee.

### Treatment and toxicity assessment

Recombinant human IL-2 (Aldesleukin, Chiron, Ratingen, Germany) was administered subcutaneously for 6 days 1 week before the operation starting with four initial high-dose injections of 10 × 10^6^ international units (IU) m^−2^ twice daily followed by a daily maintenance dose of 3 × 10^6^ IU m^−2^ until 1 day before the operation. The maintenance but not the initial dose was reduced if toxicity was encountered. Co-medication during the initial high IL-2 dose phase included the antipyretic metamizole (1 g every 4 h) and the antimemetic metoclopramide on demand. Interleukin-2-related toxicity was determined in a standardised way according to the WHO classification: mild (grade 1), moderate (grade 2), and severe (grade 3). Toxicity was monitored until 10 days after the operation.

### Immunological analyses

Peripheral venous blood was collected before and during the administration of IL-2, in both study groups 1 day before and immediately after the operation and on the first, third, fifth, and tenth postoperative day. The blood sample was drawn early in the morning after overnight fasting by puncture of an antecubital vein of the arm.

Parameters in cellular and humoral immunity, including differential blood count, T-cell markers CD3, CD4, and CD8, B-cell marker CD19, monocyte marker CD14, natural killer (NK) cell markers CD16, CD56, and CD57, activation markers CD6, CD25, CD28, CD69, and HLA-DR, progenitor cell marker CD34, and cytokine IL-1 receptor antagonist (IL-1-RA), IL-2, soluble IL-2 receptor (sIL2-R), IL-6, IL-10, transforming growth factor (TGF)-*β*1, and vascular endothelial growth factor (VEGF), were measured using standard technique and commercially available kits. *Cellular parameters:* Whole blood (100 *μ*l, EDTA) was stained with monoclonal antibodies conjugated with fluorescein isothiocyanate (Becton Dickinson, Heidelberg, Germany), phycoerythrin (Becton Dickinson), or PC5 (R-phycoerythrincyanin 5.1, Immunotech, Marseille, France). Erythrocytes were lysed by incubation with FACS lysing solution (Becton Dickinson, Heidelberg, Germany) for 15 min, and leucocyte subsets were determined by flow cytometry using a FACS Calibur (Becton Dickinson) with CellQuest Pro software (Becton Dickinson). *Cytokines:* Citrate-supplemented tubes were used and processed for plasma within 1 h. After centrifugation (3000 r.p.m., 10 min) and removal of the plasma, the samples were stored at −80°C until analysis. Cytokines were analysed using commercial quantitative enzyme-linked immunoassays (Quantikine Human Immunoassays, R&D Systems GmbH, Wiesbaden, Germany).

### Follow-up and statistical analyses

Follow-up was surveyed according to the guidelines of the European Association of Urology beginning 4–6 weeks after surgery. Data are presented as the mean±standard error of the mean (s.e.m.). Both univariate and multivariate analyses were performed depending on the data subset and question to be answered. Whenever possible, multivariate analyses were performed in order to validate univariate statistics. Characteristics of the two treatment groups were compared using the *χ*^2^-test and the Student's *t*-test for categorical and continuous variables, respectively. The primary end points were the alterations in parameters of cellular and humoral immunity. Secondary end points included toxicity, tumour-specific survival, and time to progression. Survival curves were determined using the Kaplan–Meier method and calculated from the date of nephrectomy. For univariate survival analysis, survival curves were compared using the log-rank test. It should, however, be emphasised that extensive subanalyses were limited because of the size of the patient population. Nonetheless, we address survival analysis in patients with stage IV RCC separately because of the different treatment modalities these patients received. For multivariate survival analysis, a Cox regression analysis was performed. Statistical analysis was carried out using the SPSS (version 11.5) program. In all analyses, the significance level was specified as *P*⩽0.05.

## RESULTS

Between May 1999 and September 2004, 120 consecutive patients were enrolled. Four patients were withdrawn because histology showed that they had no RCC. Therefore, the intention-to-treat population consisted of 116 patients, with 58 in each study group. The two study groups did not differ with respect to age, sex, ECOG PS, tumour stage, tumour diameter, Thoenes grade, histological subtype, location, operation time, operative technique, or perioperative blood transfusions. Table 1 summarises the patient and tumour characteristics.

### Toxicity

All patients who received IL-2 suffered from IL-2-related toxicity grade 1 (24%), 2 (67%), or 3 (9%). Toxicity subsided usually the day after the last high-dose IL-2 injection. Relevant toxicity criteria are shown in Table 2.

### Immunological analyses

**Figure 1:**
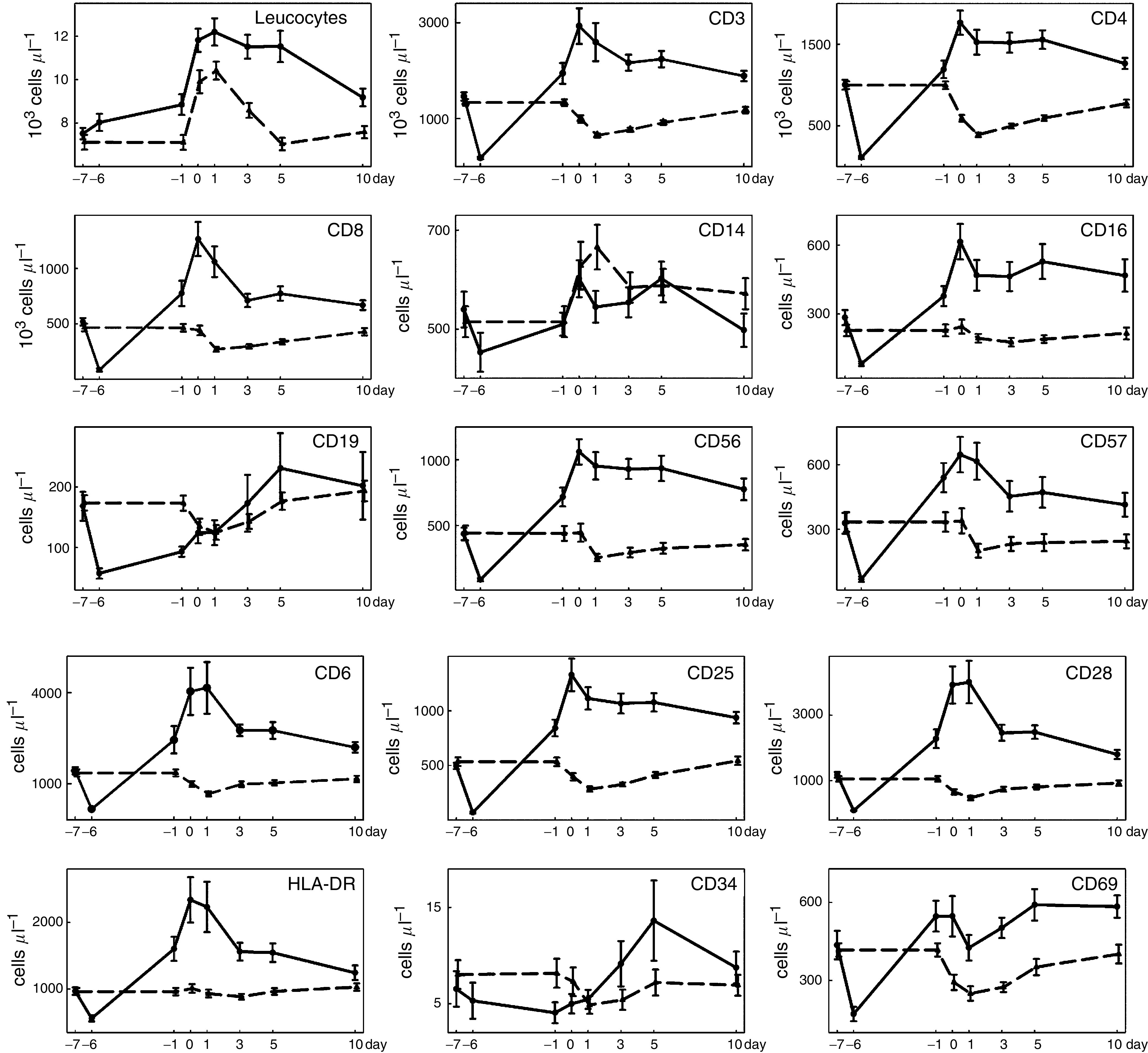
Perioperative changes of cellular markers. Mean and s.e. are indicated. (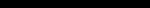): IL-2 group; (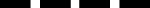): control group.

**Figure 2:**
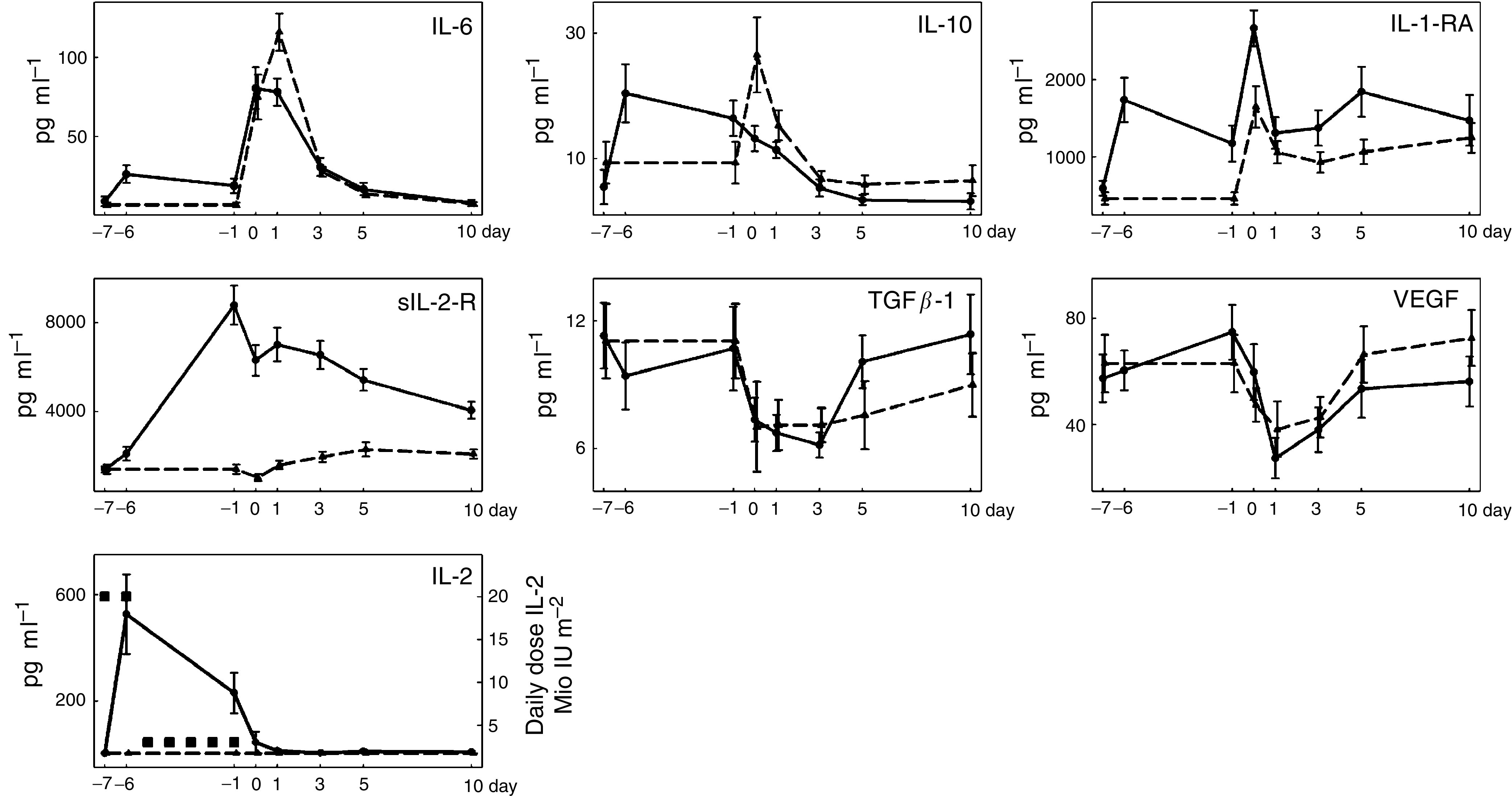
Perioperative changes of cytokines. Presentation of data as in Figure 1.

For reasons of clarity and brevity, data are presented as plots. Cellular markers and cytokines are shown in Figures 1 and 2, respectively. Mean±s.e.m. are indicated.

During IL-2 treatment, T-cell markers (CD3, CD4, CD8), activation markers (CD6, CD25, CD28, HLA-DR), B-cell marker (CD19), and NK cell markers (CD16, CD56, CD57) decreased significantly (day −7 *vs* −6), followed by an increase at the day of operation. Progenitor cell marker CD34 did not change from day −7 to day −6, but was significantly lower in the IL-2 group on day −1. In the postoperative period, all patients showed elevated leucocyte and granulocyte but decreased lymphocyte counts, T-cell markers, activation markers, and NK cell markers. However, all these alterations were significantly less pronounced in patients treated with IL-2. In contrast to their preoperative levels, the postoperative kinetics of B-cell marker CD19 and progenitor cell marker CD34 was not significantly different between both groups. Monocyte marker CD14 was significantly decreased on day −6 and day 1 in the IL-2 group.

As expected, IL-2 levels were elevated only after administration of IL-2. Interleukin-2 administration was followed by a significant counter-regulation of the soluble IL-2 receptor, IL-1-RA, IL-6, and IL-10. However, the early postoperative increase of IL-6 and IL-10 was significantly less pronounced in treated patients. No changes were found for TGF-*β*1 and VEGF during IL-2 treatment. Both cytokines showed a postoperative decrease, but were not significantly different between the study groups.

### Follow-up

At the time of analysis, 96 patients were alive, 90 of whom had no evidence of disease. The median follow-up for surviving patients was 40 months (range 1–80 months).

**Figure 3:**
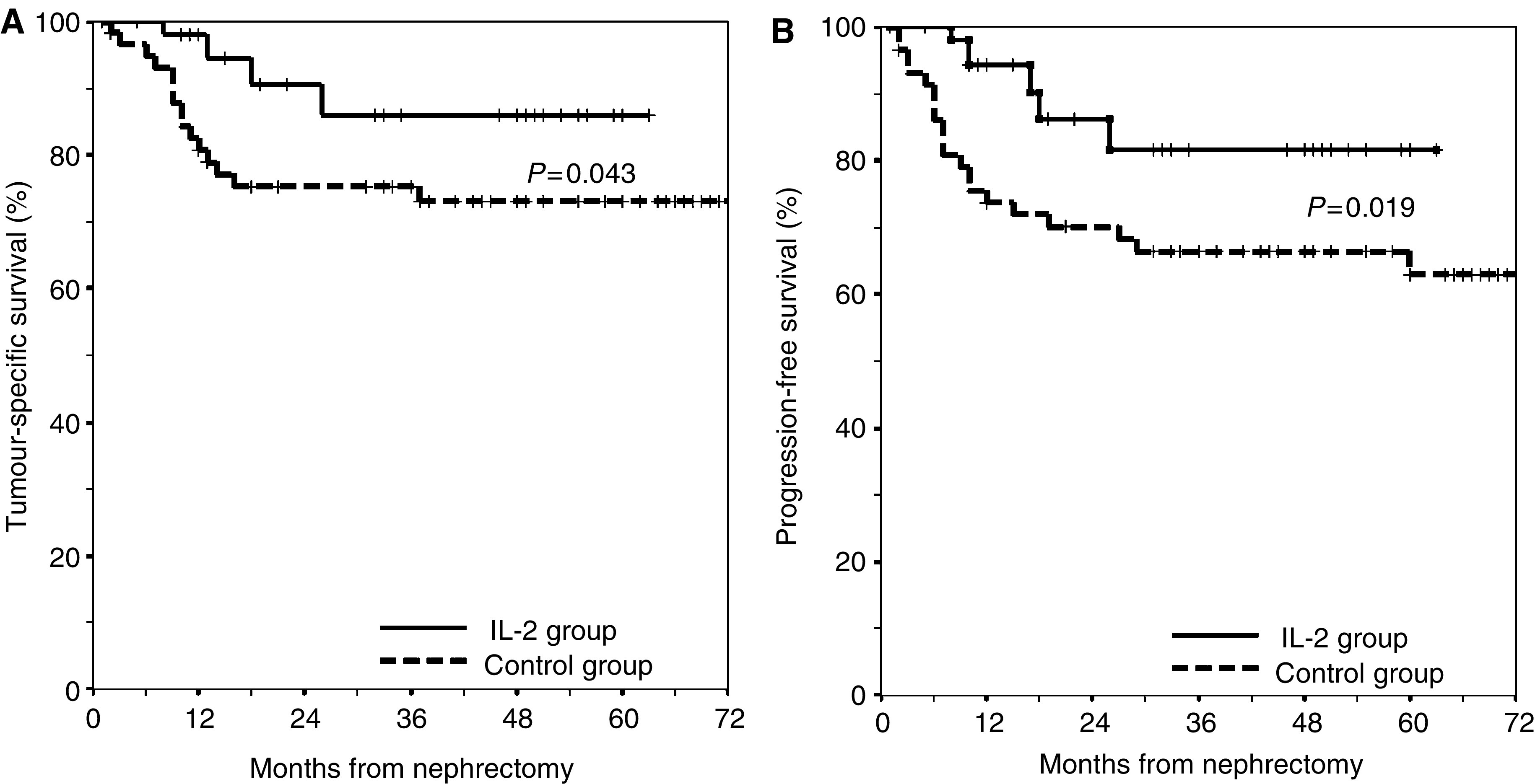
Kaplan–Meier survival estimates of patients treated with IL-2 (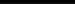, IL-2 group) and without treatment (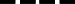, control group) according to (**A**) tumour-specific survival and (**B**) progression-free survival.

For all patients (i.e. patients of all stages), the estimated 1- and 5-year rates (±s.e.m.) of tumour-specific survival were 98% (±2%) and 86% (±7%) in the IL-2 group and 81% (±5%) and 73% (±6%) in the control group, respectively (*P*=0.043; Figure 3A). The median survival time was not reached in either study group. A similar effect was found for progression-free survival: 1- and 5-year progression-free survival rates (±s.e.m.) for patients were 94% (±4%) and 81% (±8%) in the IL-2 group, and 74% (±6%) and 62% (±7%) in the control group, respectively (*P*=0.019; Figure 3B). Median time to tumour progression was not reached in either group.

Patients with stage IV disease were addressed separately, because they received the following therapies after nephrectomy: four patients of each group immunochemotherapy (Hanover regimen: IL-2, IFN-*α*, 5-FU), four patients (IL-2 group: three; control group: one) mistletoe treatment, two patients (one of each group) surgical resection of lung metastases, one patient (IL-2 group) IFN-*α* and vinblastine, and one (IL-2 group) local immunotherapy with inhaled IL-2. The median tumour-specific survival time was 18 (±4) months in the IL-2 group and 10 (±1) months in the control group (*P*=0.015). Median time to progression was 14 (±4) months and 6 months (±1), respectively (*P*=0.006). Because of the small patient population (IL-2 group: *n*=16; control group: *n*=9), further subanalysis including a separate multivariate analysis was not meaningful.

Notably, we found no difference between the different histological subtypes with respect to perioperative immunodysfunction and survival. A more detailed statistical analysis of subgroups was hampered by the small strata size.

Multivariate analyses were performed using a Cox proportional hazard model. The following covariates were examined: age, ECOG PS, tumour stage, Thoenes grade, histological subtype, treatment with perioperative immunomodulation (yes *vs* no), leucocytes, CD3, CD4, CD6, CD8, CD16, CD25, CD28, CD56, CD57, CD69, IL-1-RA, IL-2, sIL2-R, IL-6, IL-10, TGF-*β*1, and VEGF at different time points. The only variables that reached statistical significance were ECOG PS, tumour stage, Thoenes grade, and perioperative immunomodulation. These parameters are shown in Table 3.

## DISCUSSION

IL-2 is an established and potent immunomodulating agent that exerts a striking array of pleiotropic effects on numerous target cells, the most prominent of which is on T lymphocytes (Gaffen and Liu, 2004). We showed that preoperative treatment with IL-2 effectively modulates perioperative immunodysfunction but causes only moderate short-term toxicity. The early postoperative nadir of cellular parameters of the immune system is less pronounced in IL-2-treated patients. A more detailed discussion of possible cellular mechanisms of this effect can be found elsewhere (Böhm *et al*, 2002).

Interleukin (IL)-2 modulates not only intravascular but also intratumoral immunity. Recently, Donskov *et al* (2002, 2004) found a positive correlation between numbers of intratumoral lymphocyte subsets (CD3, CD8, CD57) and both objective response and survival during IL-2-based immunotherapy. It remains to be elucidated whether intravascular rather than intratumoral immune function is critical for disease recurrence after nephrectomy.

Administration of IL-2 was started a week before surgery in order to allow the patient's immune system enough time to respond. It was discontinued 1 day before the operation so that IL-2-related toxicity did not interfere with the operation. In many immunotherapeutic regimens, IL-2 is administered over a period of several weeks. During this long period, IL-2-related toxicity is likely to accumulate, which contributes to low patient compliance. In contrast, the much shorter preoperative IL-2 regimen used here was better tolerated. Frequency and severity of side effects were within the reported range for the dosage used (Brivio *et al*, 1992, 1996). The newly introduced dose decrement after the initial four high doses of IL-2 attenuated a counter-regulation encountered in an earlier regimen (Böhm *et al*, 2002). Consistent administration of metamizole and metoclopramide increased tolerability. Possible accumulation of IL-2-related toxicity was avoided by limiting the duration of high-dose IL-2 administration to 2 days and overall IL-2 administration to 1 week. The dose decrement also enhanced the effect of IL-2 pretreatment on the elevation of T-cell, NK cell, and activation markers, and thus on leucocytes reported earlier (Böhm *et al*, 2002).

With this trial design and the timing of preoperative IL-2 administration, maximum perioperative immunomodulation was achieved in the early postoperative phase when postoperative immunodysfunction is most pronounced. The authors therefore speculate that perioperative as opposed to adjuvant immunotherapy is effective because it modulates the immune system during the most critical perioperative phase when its ability to deal with metastasising tumour cells is at a nadir.

Before the initiation of the study, some thought was spent on its design. Perioperative immunomodulation is an investigational approach and not yet a standard therapeutic option. Therefore, a phase II rather than a phase III trial was designed. A randomised phase III trial with a power of 0.80 and an alpha error of 0.05 would require more than 250 patients in each group. At the present stage, it appears that it is hardly possible to recruit so many patients, because ethical concerns may arise besides the organisational challenge. To our knowledge, no randomised trial of perioperative immunomodulation has been conducted so far on any malignancy, and the size of the verum group rarely exceeded 20 patients in published studies (Böhm *et al*, 2003). For these reasons, we opted for a well-controlled non-randomised phase II trial rather than a randomised trial. Keeping also in mind that newer therapeutic approaches focus not only on the immune system but also on other biological pathways such as the VEGF pathway and tyrosine kinase inhibition, a rather broad array of markers was assessed.

It appears that after preoperative administration of IL-2, the perioperative alterations of immunological parameters are most pronounced in the early post-operative period (Böhm *et al*, 2001). After the 10th post-operative day, these alterations are rather minor (Deehan *et al*, 1995) and can be biased by other factors. For these reasons, immunological parameters were measured during the first 10 days after surgery.

Tumour-specific and progression-free survival were longer in IL-2-treated patients. However, this study was not randomised and thus we should not over-interpret the results. We calculated the power of the trial to be 0.17. To reach a power of 0.80 and an alpha error of 0.05, we estimated that more than 250 patients would be required in each group. Therefore, we propose that to validate our survival data, future studies incorporate the approach of perioperative immunomodulation whenever possible into prospective, randomised phase III trials.

Two large studies showed that tumour nephrectomy followed by immunotherapy results in longer survival of patients with metastatic renal cell cancer (Flanigan *et al*, 2001; Mickisch *et al*, 2001). In our study, no adjuvant immunotherapy was administered, and only five of 16 patients with metastasised (stage IV) RCC received systemic immunotherapy according to departmental guidelines at the time of the trial. This does not warrant subgroup stratification. It also suggests that the survival data reported here are not corrupted by subsequent systemic immunotherapy. Tumour-specific survival in the control group is comparable with that recently reported by other groups (Motzer *et al*, 1999; Flanigan *et al*, 2001; Mickisch *et al*, 2001; Atzpodien *et al*, 2003; Verra *et al*, 2003; Atkins *et al*, 2004), suggesting that a representative and reliable patient population was assessed here.

None of the stage I patients died of recurrent disease, but two of 24 (8%) control patients and none of the IL-2-treated patients had a tumour recurrence during follow-up. Because recurrence is uncommon but does occur among stage I patients (Ljungberg *et al*, 1999; Gofrit *et al*, 2001; Pantuck *et al*, 2001; Zisman *et al*, 2002), we included these patients in our trial. Owing to the small number of events and the short follow-up, it is not feasible to assess these patients statistically.

In the early postoperative period, TGF-*β*1 and VEGF decreased, but no effect of IL-2 treatment was demonstrated. It appears that IL-2 may not interfere with newer VEGF pathway-targeted tyrosine kinase inhibitor therapies. Possibly, a combination with these therapies may be promising.

Taken together, our results indicate that preoperative treatment with IL-2 may be a feasible, yet investigational approach to complement surgical therapy and modulate some alterations of the immune system that occur perioperatively in patients undergoing tumour nephrectomy. As mentioned above, a larger randomised trial will be needed to validate the survival data.

## Figures and Tables

**Table 1:** Patient demographic, tumour, and operation characteristics

**Variable**	**IL-2 group (*n*=58)**	**Control group (*n*=58)**	***P*-value**
*Age (years)*			
Median	64	63	0.248
Range	34–81	36–84	
			
*Sex*			
Female	20 (34%)	22 (38%)	0.699
Male	38 (66%)	36 (62%)	
			
*ECOG PS*			
0	24 (41%)	29 (50%)	0.530
1	28 (48%)	22 (38%)	
2	6 (10%)	7 (12%)	
			
*Tumour* *stage (2002)*			
I	23 (40%)	24 (41%)	0.234
II	6 (10%)	4 (7%)	
III	13 (22%)	21 (36%)	
IV	16 (28%)	9 (16%)	
			
*Tumour diameter (mm)*			
Median	65	55	0.451
Range	18–200	17–150	
			
*Thoenes grade*			
G1	9 (16%)	12(21%)	0.407
G2	37 (64%)	39 (67%)	
G3	12 (21%)	7 (12%)	
			
*Histological subtype*			
Clear cell	51 (88%)	53 (91%)	0.504
Papillary	6 (10%)	3 (5%)	
Chromophobe	1 (2%)	2 (3%)	
			
*Tumour location*			
Right-sided	28 (48%)	25 (43%)	0.313
Left-sided	30 (52%)	33 (57%)	
			
*Operation time (min)*			
Median	160	165	0.864
Range	70–345	60–380	
			
*Type of incision*			
Transperitoneal	45 (78%)	42 (72%)	0.520
Flank	13 (22%)	16 (28%)	
			
*Blood transfusion, units packed red blood cells*			
Median	0	0	0.516
Range	0–6	0–6	

IL-2=interleukin-2; ECOG PS=Eastern Cooperative Oncology Group performance status.

**Table 2:** Interleukin-2-related toxicity according to WHO

	**Grade 0**	**Grade 1**	**Grade 2**	**Grade 3**	**Grade 4**
Haematological	58	0	0	0	0
					
*Gastrointestinal*					
Bilirubin	58	0	0	0	0
Transaminases	27	14	12	5	0
Oral	53	4	1	0	0
Nausea/vomiting	41	9	8	0	0
Diarrhoea	55	3	0	0	0
Constipation	53	5	0	0	0
					
*Renal*					
Blood creatinin	43	13	2	0	0
Pulmonary	58	0	0	0	0
Fever with drug	9	15	34	0	0
Allergic	58	0	0	0	0
Cutaneous	9	31	18	0	0
Hair	58	0	0	0	0
Infection	57	1	0	0	0
					
*Cardiac*					
Hypotension	52	0	6	0	0
Neurotoxicity	58	0	0	0	0

**Table 3:** Cox proportional hazard model

**Covariate**	**Hazard ratio (95% CI)**	***P*-value**
ECOG PS	0.099 [0.020–0.495]	0.005
Tumour stage	0.043 [0.007–0.254]	0.001
Thoenes grade	0.317 [0.120–0.837]	0.020
Immunomodulation (yes *vs* no)	0.165 [0.059–0.463]	0.001

CI=confidence interval; ECOG PS=Eastern Cooperative Oncology Group performance status.
